# Patients' Participation as It Appears in the Nursing Documentation, When Care Is Ruled by Standardized Care Plans

**DOI:** 10.5402/2011/707601

**Published:** 2011-07-10

**Authors:** Christina Andreae, Mirjam Ekstedt, Ingrid Snellman

**Affiliations:** ^1^Department of Medicine, Mälar Hospital, 631 88 Eskilstuna, Sweden; ^2^Research and Development Center/Center for Clinical Research, Sörmland County Council, 632 20 Eskilstuna, Sweden; ^3^Department of Health Care Sciences, Ersta Sköndal University, P.O. Box 11189, 100 61 Stockholm, Sweden; ^4^School of Health, Care and Social Welfare, Mälardalen University, P.O. Box 325, 631 05 Eskilstuna, Sweden

## Abstract

This study aimed to describe inpatients with myocardial infarction and their participation in care as documented in the nursing records when standardized care plans are used in care. The use of standardized care plans not only has increased the quality of medical treatment but has also overlooked patients' opportunities to participate in their own care. There is a lack of knowledge about how standardized care plans influence patients' participation in nursing care. Data were collected from thirteen patients' records with diagnoses of myocardial infarction. Participation in the decision-making process and participation associated with “sharing with others” were searched for in the analysis. The analytical process was guided by content analysis. The findings were grouped into two categories: patients' intermediary participation and patients' active participation. The main results indicated that patients' intermediary participation depended on healthcare professionals' power to rule the nursing care situation.

## 1. Introduction

Standardized care plans have been in place since the middle of the 1980s and are used in several countries with the objective of achieving a quality-assured standard of care [1–3]. Standardized care plans make it possible to give good care to everyone regardless of who is caring, and standardized care plans are particularly important for quality of care [4]. Standardized care plans are both health and cost effective [5, 6] although there are few studies that show how standardized care plans affect patients' participation and their influence on health care. Which conditions are required of the patients in order to participate in nursing care and treatment, if care is already planned before the patient's admission to hospital? This study presents the findings of patients with myocardial infarction and their participation in care, as it appears in the nursing documentation.

When standardized care plans are used in nursing care, the care plans are designed in advance for a specific patient group. The standard care plan lists the treatment steps to be performed as well as the times [4, 7–10]. The effect of standardized care plans on different outcomes is wide and has been used in care of patients with different diagnoses. For example, patients with heart failure who received medical care with guidance of a standardized care plan showed a decrease in mortality from 14% to 7% [11]; for patients with pneumonia, mortality decreased from 10% to 5% [12]. Another outcome using standardized care plans is that fewer postoperative complications occurred. For patients undergoing hip and knee replacement, the reduction of complications including infections and deep vein thrombosis were 4% for the patient group as standardized care plans were used opposed to 13% for the control group. Among patients undergoing colon resection, a reduction in complications of 13% was noted [13]. There is no doubt that standardized care plans contribute to a better quality of medical care and almost to a lower cost [14, 15], but how does standardized care plan affect the patients' ability to participate in nursing care?

Patient participation is considered a basic condition for good care [16], and the term is common in European countries [17]. Health care personnel are responsible for ensuring that patients have the opportunity to participate in care related to their individual needs, wishes, and desires [16, 18]. Nurses are responsible for ensuring that nursing care is designed and implemented in consultation with the patient [19, 20]. Above all, patients' own desires should be considered when nurses give priority to health measures in their nursing care. All information concerning planned and performed care, health history, and body functions as well as self-perceived problems have to be written in the patient record [21].

The term “patient participation” can be referred to partaking in decision-making process in matters pertaining to health. Participation relates to decision-making regarding one's treatment and the right to be informed [22]. For nurses, patients' participation means to protect patients' privacy and allow them to be involved in their own care [21]. Participation from nurses' view is also about patients' need for knowledge regarding living with illness and medication in order to influence their own care [23–25].

The term “patient participation” can also be associated with “sharing with others” in some action or matter, taking part in an activity, or being involved in a life situation [22]. Patients can experience participation when they talk about their symptoms or when nurses listen and show respect for the wishes that come up in conversation [26]. Participation from the patients' view is also about the information that nurses provide when it is based on patients' own needs [26–28]. Knowledge makes it easier for patients to be involved in care [29] provided that the information is adapted to and based on the patients' level of knowledge [30–32]. 

It appears that patients are quite often dissatisfied because the information provided is general and not based on each patient's expectations [26]. If the patient is treated as an individual, then they might have greater opportunities when they are sick as to how they can be involved and what they can do [29, 33]. Today, nurses frequently discuss the standardized care plans in relation to patients' participation, and many questions are unanswered, probably because it has not been studied very well. Knowing more about how standardized care plans affect the patients' engagement in their own care is crucial. Therefore, this study focused on patients' participation in nursing documentation when the standard care plan rules the care.

**Aim**

The aim was to describe participation in care of inpatients with myocardial infarction as written by the nurses in the nursing records when standardized care plans are used in care.

## 2. Materials and Methods

### 2.1. Design and Setting

Current study is a qualitative descriptive study, using open-ended questions to guide data extraction from nursing records. The study was performed at a medium-sized Swedish hospital, unit for coronary heart inpatients. Based on previous research on patient participation in care, the following two aspects of participation have been searched for in the nurses documentation in the patient records: (a) patients' participation in nursing care associated with the decision-making process affecting the patients' health and lives, for example, “freedom to make choices and to take responsibility for their health” and (b) patients' participation in nursing care associated with “sharing with others,” for example, “talk about their symptom and thoughts.”

### 2.2. Data Collection

A consecutive series of patient records was selected. Inclusion criteria for the study were (a) patients with myocardial infarction, (b) care according to a standardized care plan, and (c) patients who were able to speak and understand Swedish. The selection was conducted in two steps. Step one was to collect a patient data list based on the following criteria: diagnosis myocardial infarction, admission in hospital for treatment in a coronary care unit and also an admission to hospital from 11-15-2007 to 02-15-2008. The initial sample revealed a patient data list including a total of 54 patients with full name and date of birth. Step two was to locate and read each of the medical records and select those who had been cared for according to a standardized care plan, who had a length of stay between 2 and 5 days which was comparable to standard care plan, and who were able to speak and understand Swedish. Finally, the nursing documentations was printed out from the medical records. The selected documentation involved the following areas: nursing history, nursing status, nursing diagnoses, nursing goals, nursing intervention, nursing outcome, and nursing discharge notes. There were thirteen nursing records matching the study inclusion criteria and the aim of the study.

### 2.3. Ethical Consideration

Permission for the study was obtained from the master of hospital clinic. All records were managed according to the Swedish data inspection board requirements [34]. Confidentiality was assured for both patients as well as the nurses who had written the records. The identifying information was not available to anyone who was not directly involved in the study. Other not significant data from the copy of the nursing records was removed and destroyed.

### 2.4. Data Analysis

Nursing records were analyzed for describing how patients' participation emerged in nursing documentation, when a standardized care plan was used in care. Two aspects of participation have been searched for in the analysis: (a) patients' participation in nursing care associated with decision-making process affecting their own health and (b) patients' participation in the nursing care associated with “sharing with others.” The documentation of participation was transcribed, and the analytical process was guided by content analyses, as described by Graneheim and Lundman [35]. Content analysis is an interpretative process where the context is taken into account. The aim was to describe what the text said concerning patients' participation, without a deeper interpretation [35, 36]. The analysis was performed in several steps. Initially, the nursing record was read several times to obtain a comprehensive picture about the documentation of patients' participation. Secondly, words and sentences related to each other by content and context concerning patients' participation were broken into meaning units by the first author (C. Andrea). Without losing the contents of the text, meaning units were condensed and labeled with codes at a low level of abstraction. After rereading and understanding the participation of the codes, they were sorted into preliminary categories representing similarities and differences according to the two aspects of participation. Finally, the preliminary categories were formulated as categories and subcategories according to the manifest content at a higher level of abstraction. During the analysis process, the movement took place between the whole and parts of the text to ensure that the interpretations were made on a higher level of abstraction. To ensure trustworthiness, a tentative border was discussed between the authors and revised several times until a unified understanding was reached. To increase the transparency of the interpretation, categories and subcategories are illustrated with quotations.

## 3. Results

Participation in care of inpatients with myocardial infarction as written by the nurses in the nursing records when standardized care plans are used in care is contained through two categories underlying their subcategories. Category *patients' intermediary participation* emerges through subcategories *self-perceived problems, body experiences, *and* barriers to participate in care*. Category *patients' active participation* emerges through the subcategory *self-determination.*

### 3.1. Patients' Intermediary Participation

The analysis indicated an intermediary participation when nurses assessed patients' narrative of their symptoms by their character and intensity in an emotional chaos and when patients are told about how bodily function affected their lives. Thoughts around the life situation of being sick and unpredictable future were described with concern and anxiety. The analysis also indicated that intermediary participation depended on external reasons when relatives and nurses affect patients' involvement in their own care.

#### 3.1.1. Self-Perceived Problem

Patients' self-perceived problem was described in the nursing records as unpleasant feelings in the breast, when the feelings came suddenly and were not recognized. These problems were characterized by pain and a tightening in the chest with spreading over the chest area. Several symptoms of feelings such as palpitations, difficulties in breathing, and a feeling of weakness lead patients to seek medical care. These symptoms arise in connection with physical activity. When breast pain was triggered by activity, on admission the patients made a mental note. The pain's intensity in the chest was described through statements about the degree of pain: from no pain to mild pain or duration of pain; the pain comes and goes often with physical activity.

In the analysis emerged other self-perceived problems when the nurses informed about the health situation and the planned care. Patients suffering from myocardial infarction were emotionally affected and expressed concern about an unpredictable future. Patients found that the heart attack was a threat to survival. Fear of death was apparent by the fear of exposing themselves to examinations that they could not control. To lose control of one's life was a shocking experience and a disappointment. A nurse documented the following in the nursing journal: “Have received verbal information about myocardial infarction and angina. Patient thinks it feels good even though he is shocked that it was a heart attack” (G2c). The changed life situation was expressed by a constant fatigue and lack of energy with difficulty to absorb information about the care and treatment but was also expressed through psychosomatic problems.

#### 3.1.2. Body Experiences

Patients' experiences of their own bodies were expressed through stories about bodily functions that affected their daily lives. The analysis revealed that sensory functions, oversensitivity, elimination, activity, and sleep are key parts of the patients' bodily functions.

The patients' visual and hearing impairments and their need for help were reported in nursing records. Eyeglasses and hearing aids were described as the most common aids. Oversensitivity was mainly seen with milk, mold, pollen, and drug-induced oversensitivity. One nurse documented in a nursing journal: “Describes that she has had problems with contrast, but does not swell up” (G3a). Furthermore, patients recounted tales of elimination which mainly related to frequency of urination and defecation which did not follow any specific pattern. Ability to walk and move in daily life was related to insecurity with fear of falling while reduced physical functions were related to the inability to participate in rehabilitation after myocardial infarction. There were also notes in the documentation that sleep was irregular, ranging from uninterrupted sleep throughout the night to a couple of hours. The sleep disturbances had been present for a long time, and they carried it with them into the new hospital environment.

#### 3.1.3. Barriers to Participate in Care

The analysis indicated that nurses and relatives affect patients' abilities to participate in care: “Discharge today or tomorrow depending on how the wife feels the patient's condition” (G1b). Relatives' needs to check the care patients receive and nurses information to relatives affect the patients in a negative way. That is to say that they themselves were at the center but stood outside their own care and treatment. Patients' ability to be central in their own care and treatment was hindered by the involvement of relatives. The involvement came out during discharge with the responsible doctor and nurse. Relatives received information about the current care and treatment, which contained information about medical treatment concerning invasive coronary investigation and pharmacological treatment. The relatives' wish to decide on the care and treatment meant that nurses had repeated telephone conversations with them. Discussions focused on the health status related to each patient's ability to support themselves after discharge from hospital and planning of home care services. The nursing documentation showed that the relatives' decision was critical at the time of care planning and when the patients could be discharged from the hospital.

### 3.2. Patients' Active Participation

The analysis highlights the patients' *self-determination* through knowledge, freedom to make choices, and wishes. Patients reflected over the care and treatment that they received, because they wanted to know more about how the treatment affects future recurrence. The analysis indicated also that the patients were autonomous and self-supportive persons taking responsibility of their own lives when taking active part in seeking information.

#### 3.2.1. Self-Determination

Patients' physical needs were central, and the routine nursing measures sometimes had to take second place. The nurses showed the way for different care actions, but it was the patients who ultimately decided which nursing measures would be accepted or rejected. The patients' wish for autonomy through need for knowledge and desire to make their decisions came out in the nursing journal.

In order to move forward in life after myocardial infarction and to be able to change their lifestyle, the patients searched for knowledge about their disease. The need for knowledge was illustrated by their participation in secondary prevention programs. Patients also asked questions when it was all over and when it was time to reflect. Questions could be about the treatment of coronary artery disease, such as the stents inserted in coronary arteries.

Changes in dietary practices and reduced physical activity during hospitalization affected patients' blood sugar levels. Analysis of nursing records showed that patients with diabetes preserved their ability to take their own decisions about insulin doses. When blood glucose levels fell, the patients decided themselves on which insulin levels were most suitable. The nurse documented the following: “Feels low blood sugar, to take another check before she goes to sleep tonight, check your blood sugar yourself. Blood glucose 9.8 mmol/L gives regular insulin dose in consultation with patient. Blood glucose at 11 mmol/L, she takes half the dose of insulin and eats a sandwich” (G3b).

There was information in the records that suggested that patients took their own decisions about participation in secondary education on heart disease prevention. The analysis showed that patients' prior knowledge of heart disease was a basis to participate or to refrain from cardiovascular school. The analysis also showed that patients' previous knowledge led to them refrain from receiving nursing information on the disease. Patients' own decision was all about waiving routine care and asking for treatment that was not routine, for example, to want to refrain from peripheral venous catheters because of pain and discomfort, or regarding nicotine addiction, stress and pain to express a willingness to get help in the form of medicine.

## 4. Discussion

The study revealed that patients' participation in nursing care was intermediary and active. The category *intermediary participation* is established through the subcategories *self-perceived problems*, *body experience*, and *barriers to participate in care*. The category active participation emerged through the subcategory self-determination.

An intermediary participation is viewed in light of the nurses' assessment of patients' self-perceived problem as pain, breathing, sudden unpleasant feeling in the chest, and assessment of body experiences. Thoughts around the life situation of being sick combined with a feeling of insecurity and anxiety for the future were described in nursing records. In accordance with current Swedish regulations [37], nurses' assessment about symptoms and health situation followed the nursing process to ensure quality of care which the nurses documented in the patients' medical records. However, from the patients' point of view, this may lead to negative consequences in a situation where patients only answer questions and are not involved in decisions related to unique needs and circumstances.

Previous studies show that patients' engagement and reflection in communication is an important issue for shared decision-making [27, 28, 38]. Nurses sometimes avoided this reflection on problems [39] which influenced the patients feeling of not being seen and not having the opportunity to participate [40]. When nurses in the current study informed about diagnosis, patients described feelings of insecurity and anxiety about the future, leading to tiredness and lack of energy. These emotional problems were probably left unanswered because nurses did not describe in the nursing records how these emotions were supported, which decreased the patients' ability to participate in care situations. One reason to avoid reflection can be that nurses are focused too much on the procedure of standardized care plans. However, there might be a gap between what nurses describe in the journal and how they support patient's reactions of anxiety about the future, tiredness, and lack of energy in reality. We simply do not know anything about the content of the conversations only what the nurses describe in the records.

Standardized care plans facilitate nursing care because the nurses will be reminded about nursing actions that are important for ensuring quality care. According to Grimen [41] routines, for example, standardized care plans facilitate nursing care, but there is a risk that nurses focus too much on nursing measures in the standardized care plan and do not perceive other needs expressed by the patient. 

The study also indicated that intermediary participation depended on external reasons when nurses and relatives plan and decide for the patients' own care. Relatives assumed responsibility for the planning of care and decided the time for discharge from hospital. According to Sahlsten et al. [40], relatives hindered the patients in participating because they decided over the patients' heads. Relatives also acted in an overprotective way that influenced the feeling of being uncertain about the future [30, 42]. In Sweden patients and relatives have to be involved in care on the basis of health condition and concerning mental state, even concerning the planning of discharge [18, 43]. Nurses in the current study acted in a non-patient-centered way when information about discharge in the first instance was dedicated to relatives and if the patient received that information is still unclear. When deciding on behalf of somebody else, nurses have an impact on the opportunity of a person to participate in their own care [41]. Reasonably, we cannot say that patients were excluded or ignored to participate and there is no evidence in this study that standard care plans hindered patients' participation. According to a study with focus on elderly inpatients and their experiences about participation in clinical decision-making, nurses decided what they thought was best, but not in congruence with the patients' preferences [44].

Patients' participation increases when information about illness is based on their own needs [27, 28] and in front of earlier knowledge [30–32]. In the current study patients' participation tended to be intermediary because the nurses' information about diagnosis and treatment is guided by standardized care plans. Most information came from verbal and written information which runs the risk leading to insufficient information concerning individual needs, and this influenced patients in feeling that they were not participating. In line with earlier studies, general information indicates problems concerning lack of individuality [45–47]. In contrast Pollock et al. [48] found that patients with cancer receiving standardized care plans are more active in participation when verbal rather than written information is received because of the opportunity to face questions and anxiety concerning diagnosis and treatment. In the present study it is important to consider that verbal information was possibly grounded in a deeper conversation, but in this case it was not visible in patients' records. It appears that nurses' approaches to deliver information are more concerned with following standardized care plans than with communication with patients. On the other hand, it is well known that nurses have problems in communication and inviting patients to participate in nursing care due to uncertainty and insufficient control over care situations [23, 24]. Benner et al. [49] declare that the situation often depends on nurses' experiences of the caring profession.

An active participation is viewed in light of the nurses' assessment of patients' *self-determination* through knowledge, freedom to make choices, and wishes. Patients asked questions and reflected over the care and treatment that they received because they wanted to know more about how the treatment affects future recurrence. According to Zoffmann et al. [39], reflection and increased knowledge help patients create opportunities to make their own decisions.

The study indicated the patients as autonomous and self-supportive persons taking responsibility for their own lives when taking an active part in seeking information. Furthermore, it is possible that standardized care plans facilitated nurses in having more time to care which is consistent with Dahm and Wadensten [50] and their study showing that both documentation time and duplication are reduced.

An important issue concerning self-determination is patients' decision-making about treatment, for example, when nurses measure blood test samples to check for diabetes leading to discussions about the results and treatment with patients. This is from the patient perspective as patients make decisions themselves about both the treatment and even followup instead of allowing nurses just to give an insulin dose according to the medical record. Patients thus become active as experts about their own illness with the power to rule the care situation irrespective of living in a foreign environment in hospital. Moreover, patients' active participation in decision-making in this study can be related to self-determination with the freedom to make choices in nursing care concerning their own health and treatment. It is clear that nurses' caring support from standardized care plans does not obscure patients' participation to make choices. For patients it is important that the care provider is skilled in giving sympathy and that they ask patients about their own wishes [51].

During admission, patients coping with pain, craving for nicotine and stress, were a fundamental concern regarding self-determination. Patients in the current study were focused on receiving help and clearly required medical treatment and it is essential to view their own will. There are clearly questions behind what motivates the patients to be active in participation. Similar symptoms before hospital, for example, pain, craving for nicotine and stress, may be an influence for patients asking for medical help because they have probably experienced these symptoms before and they know how to manage them. Moreover, the standardized care plan describes that nurses should encourage patients to ask for help in any cases during hospital admission, not only in relation to cardiac events.

## 5. Methodological Considerations

This methodological approach was appropriate for the aim of the study. However, there are a number of limitations when using secondary data. First, data was collected from nurses' documentation and the text content is dependent on nurses' experience of documentation, professionalism, meeting with patients, nurses' involvement in the patients' health and needs, experience, and workload in the department. Exactly what determines the content in documentation also depends on subjective judgments about what nurses believe is important for the care. These judgments result in an imbalance on how participation will emerge and be managed in each specific patient record as well as in clinical practice, and this point of view needs to be expanded. Second, it is difficult to assess the patient's involvement in nursing care when standardized care plan is used due to the limited documentation in nursing records. It is well known that documentation is limited in nursing records; despite this our study shows some results about patients' participation in nurses' documentation. The reason for this may depend on the fixed structure of the care plan, which guides the nurses to ask questions about their participation. In the future the standardized care plans might be seen more as decision aids, supporting patients to be involved in their care and treatment which in turn will be documented in the nursing records.

## 6. Conclusion

Patients' participation as it appears in the nursing journal in this study is intermediary, which is shown by nurses' focus on nursing process, patients' narratives, and the relatives' involvement in care and treatment. To the patient, intermediary participation means a care managed by others and not always based on personal desires and needs, a care thus deviating from descriptions of how care should be planned and implemented. Intermediary participation assumes some degree of autonomy, taking responsibility for one's own care based on experience of the health problem. However, it is important to face patients' view of participation when care is ruled by standardized care plans and this study needs support from others to investigate this.

## 7. Relevance to Clinical Practice

This study shows how important it is that nurses have knowledge about how standardized care plans may hinder patients' involvement in care. Nurses use the standardized care plan to give patients a quality-assured standard of care, but there is an imminent risk of nurses focusing too much on following the standard care plan and not understanding what the patients really express. When care is ruled by a standardized care plan, nurses need to be sensitive to and recognize the individual patient's thoughts and reflections about their situation to be sick, there also needs to be room so that patients are able to reflect and discuss their concerns with a nurse. There is a need for nurses to tell patients that care is governed by a standardized care plan and that this may need to be adjusted according to patients' wishes and needs. In this way, standardized care plans serve as a resource; in other words, they allow patients to be involved in the care rather than being a hindrance.

##  Conflict of Interests

There are no significant financial or personal interests in the products, technology, or methodology in the paper.

##  Authors' Contributions

Study design was conducted by C. Andreae, M. Ekstedt, I. Snellman; data collection and analysis by C. Andreae, I. Snellman, and paper preparation by all of the authors.
